# Predictors of ≥50% volume reduction after radiofrequency ablation of uterine fibroids: a single-center retrospective case series in Romania

**DOI:** 10.3389/fmed.2026.1710087

**Published:** 2026-03-17

**Authors:** Viorel-Aurel Suciu Lazar, Andreea Sotoc

**Affiliations:** Department of Obstetrics and Gynecology, Spitalul Pelican, Oradea, Romania

**Keywords:** heavy menstrual bleeding, hysteroscopy, leyomiomas, minimally invasive gynecological techniques, radiofrecuency ablation, uterine fibroids

## Abstract

**Study objective:**

The study aimed to explore the potential of routinely collected pre-procedural clinical and ultrasonographic parameters (age, classification, baseline fibroid volume, and baseline Doppler score) in predicting treatment response following transvaginal radiofrequency ablation (RFA). We employed a combined machine learning-based feature ranking and regression interpretability approach to provide a proof-of-concept for future data-driven predictive models.

**Design:**

This study was designed as a single-center retrospective case series.

**Setting:**

The study was conducted at a public hospital in Romania, where RFA was introduced as a minimally invasive alternative for uterine fibroid treatment.

**Patients:**

Twenty-two fibroids were treated in women aged 28–52 years (mean age 38), who were selected based on the presence of symptoms or documented fibroid growth.

**Interventions:**

Transvaginal ultrasound-guided radiofrequency ablation was performed using the VIVA RF system. Baseline and post-procedural measurements included fibroid size and volume, vascularity as measured by Doppler score, and anatomical classification according to FIGO criteria.

**Measurements and main results:**

Fibroid volume decreased from a mean of 60.82 cm^3^ to 28.3 cm^3^ (−54.0%), while the Doppler score decreased from 3.59 to 1.41 (−59.8%). An exploratory feature-based analysis using four pre-procedural variables (age, FIGO classification, baseline fibroid volume, and baseline Doppler score) was conducted as a proof-of-concept, highlighting the challenges of robust modeling in small cohorts. In the multivariable logistic regression analysis, no statistically significant association was observed between treatment outcome and patient age or FIGO classification.

**Conclusion:**

This proof-of-concept study demonstrates the potential of using routinely collected pre-procedural data for predictive analytics in RFA of uterine fibroids. The successful outcomes observed in two submucosal fibroids (G0 and G1) suggest that RFA followed by hysteroscopic myomectomy may serve as a viable two-step fertility-preserving treatment approach. However, the small sample size and class imbalance highlight the critical need for larger, prospective, multicentric studies to develop clinically valid predictive models for minimally invasive fibroid treatment.

## Introduction

Leiomyomas, commonly referred to as uterine fibroids, are the most prevalent benign tumors in gynecology, affecting up to 70% of women by the age of 50 (1). Accurately determining their prevalence remains challenging, as many fibroids are asymptomatic and are often discovered incidentally during routine pelvic examinations or imaging studies performed for unrelated reasons (2).

Research conducted in the United States highlights a notable racial disparity in fibroid incidence (3). By age 35, approximately 60% of African-American women are affected, with the rate increasing significantly by age 50. In contrast, among Caucasian women, the incidence is approximately 40% at age 35 and rises to nearly 70% by age 50.

Histologically, uterine fibroids represent a monoclonal proliferation of a single smooth muscle cell originating from the myometrium, accompanied by variable amounts of extracellular matrix components such as collagen, fibronectin, and proteoglycans (3, 4). Substantial evidence supports the hormone-dependent nature of fibroid growth, as they typically arise during reproductive years and tend to regress after menopause (3).

The most frequently reported symptom is abnormal uterine bleeding, which may or may not be associated with the menstrual cycle (5). Symptom severity often correlates with the fibroid’s location relative to the uterine cavity. Other functional manifestations may include pelvic pressure, lower back or abdominal pain, dysmenorrhea, increased urinary frequency, and constipation, often due to the compression effect of large fibroids. Uterine fibroids may also adversely affect fertility and pregnancy outcomes, contributing to infertility or pregnancy complications, such as preterm labor, cesarean delivery, antepartum hemorrhage, fetal malpresentation, and growth restriction.

To determine the most appropriate therapeutic approach, accurate mapping of the position, size, and appearance of uterine fibroids is essential, and the primary imaging modalities employed for this purpose include ultrasound, saline-infusion sonography, and magnetic resonance imaging (MRI). Ultrasound remains the most commonly utilized technique due to its widespread availability. Ideally, both transabdominal and transvaginal scans should be conducted, as the transvaginal scan offers superior sensitivity and specificity in detecting small fibroids, even those as small as 5 mm in diameter (4).

Regarding treatment strategies for uterine leiomyomas, both pharmacological and interventional options are available. Medical therapies often include various hormonal treatments or hemostatic agents such as tranexamic acid, while the more invasive approach includes hysterectomy, myomectomy, and uterine artery embolization (2).

Despite the range of therapeutic possibilities, patient adherence to these treatments may be limited by the growing preference for minimally invasive solutions (6). In response to this trend, radiofrequency ablation (RFA) has emerged as a viable alternative to more invasive procedures, demonstrating encouraging outcomes. RFA involves the application of radiofrequency energy via one or more electrodes, leading to coagulative myolysis of the fibroid tissue (7). This technique can be deployed laparoscopically, transabdominally, or, most frequently, transvaginally (7).

While the majority of the studies published to date focus on volume reduction and relief of symptoms, there is scarce evidence of a systematic Doppler assessment of fibroids before and after radiofrequency ablation using a standardized and reproducible score. There is also no solid evidence in the published literature regarding the age of the patients treated by RFA, accurate mapping of fibroids before and after RFA, or using a combination of pre-procedural predictors to determine a favorable response.

In Romania, radiofrequency ablation of uterine fibroids was introduced only in recent years and is still practiced on a low scale. Our unit was the first to provide this procedure in a hospital setting.

The primary aim of our study was to demonstrate the feasibility and potential of collecting simple, routinely available pre-procedural clinical and ultrasound data, such as patient age, fibroid dimensions and volume, Doppler vascularity score, and FIGO location, to build preliminary predictive models for treatment response after radiofrequency ablation (RFA). By employing a hybrid methodological framework that combines Random Forest feature importance ranking with logistic regression analysis, this study evaluates the relative contribution and directional impact of these parameters on achieving a ≥50% volume reduction. Ultimately, this study serves as a proof-of-concept to guide future, larger-scale data collection efforts and the development of more robust decision-support systems in minimally invasive gynecologic therapy.

## Materials and methods

This case series included 22 fibroids. The mean age of the patients was 38 years (range: 28–52 years). RFA was performed as a singular procedure, with no repeat interventions, spanning over a period of 11 months (April 2024 to March 2025), and follow-up visits were conducted for up to 14 months after the initial procedure.

### Patient selection

Indications for treatment included abnormal uterine bleeding in 8 patients (heavy menstrual bleeding or postcoital bleeding), a significant increase in fibroid volume (≥50%) over the preceding 12 months in 5 patients, and infertility in 3 patients. The exclusion criteria were consistent with those commonly applied for radiofrequency ablation and included pregnancy, active infection, malignancy, absence of symptoms or lack of desire for future pregnancy, and stable fibroid size and appearance over time.

### Ultrasound assessment

Fibroid position was determined using the FIGO classification, and for the Doppler score, we used a classification from 1 (no blood flow) to 4 (very high blood flow), as described by the Morphological Uterus Sonographic Assessment (MUSA) group (8) in Table 1. To ensure reproductibility, ultrasound machine settings were standardized in accordance with Frijlingh et al. (9).

**Table 1:** MUSA criteria used for Doppler assessment of uterine fibroids as published by Van Den Bosch et al. (8).

Score	Color Doppler appearance	Description
1	No detectable vascularity	Complete avascularity
2	Minimal vascularity	A few scattered color signals
3	Moderate vascularity	Several color signals, but not dense
4	Abundant vascularity	Dense and continuous color Doppler signals

### Radiofrequency procedure

As part of the RFA protocol in our unit, we routinely used the following premedication: antibiotic prophylaxis (Cefuroxime 1.5 g) and dexamethasone (8 mg).

Radiofrequency ablation was performed using the VIVA RF system manufactured by StarMed with adjustable-tip electrodes (0.5–30 mm) in continuous mode. Depending on the fibroid size, volume, and location and the technique used (moving-shot or single-shot), the energy deployed varied between 60 and 140 W. The time used for shots ranged between 15 and 120 s. All procedures were performed transvaginally under ultrasound guidance using Voluson E8 (General Electric) with an IC5-9D endocavitary probe. Each procedure was completed when more than 80% of the fibroid exhibited increased echogenicity and the absence of blood flow within the fibroid was confirmed using Color Doppler or Power Doppler.

In three cases, a biopsy was performed prior to radiofrequency ablation using a TruCut needle due to a slightly atypical ultrasound appearance of the fibroids, but all three were subsequently confirmed as leiomyomas by histological analysis.

### Data collection and statistical analysis

A dual-stage analytical framework was adopted to evaluate the potential of routine parameters in predicting treatment success, defined as a fibroid volume reduction of ≥50%. Random Forest was used as an exploratory feature-ranking tool to identify the pre-procedural variables most strongly associated with treatment response. This machine learning approach allowed for the evaluation of each feature’s relative contribution using the gain metric, producing a hierarchical ranking of predictors. Subsequently, the multivariable logistic regression analysis was applied to provide clinically interpretable effect estimates (odds ratios) for these features, ensuring a robust associative analysis alongside the predictive modeling. This transition was implemented to quantify the independent effect and direction of association for each predictor, which complements the non-linear insights provided by the Random Forest model. Given the small sample size and ordinal nature of certain variables (FIGO stage and Doppler score), these variables were included as numeric predictors in the regression models to maximize statistical power.

## Results

A total of 22 fibroids were ablated: 1 G0, 1 G1, 4 G2, 12 G3, and 4 G4 fibroids. The characteristics and outcomes of these fibroids are listed in Table 2.

**Table 2:** Characteristics of patients included.

Fibroid	Age	Gesta	Para	Position (FIGO)	Size before RFA (mm)	Volume before RFA (cm3)	Doppler score before RFA (MUSA)	Complaints	Intention of getting pregnant	RFA procedure	Size after RFA (mm)	Volume after RFA (cm3)	Follow-up visits after RFA (days)	Doppler score after RFA (MUSA)	Complications
1	40	2	1	G3	61.3/53.9/65.3	112.9	4	Volume increase (from 64.5 cm^3^ to 112.9 cm3 in 12 months)	No	03.10.2024	66.0/60.5/65.9	137.7	26	2	No
											57.4/50.9/55.7	85.2	105	1	
2	43	0	0	G3	49.9/44.6/47.2	51.9	4	infertility	Yes	29.11.2024	40.1/40.3/38.6	32.6	49	1	No
											41.1/39.7/34.9	29.8	104	1	
				G3	30/36/33	18.6	2				26.7/30.3/23.5	9.9	49	2	
											26/30.3/21.3	8.8	104	1	
3	28	0	0	G3	64.1/40.6/62.4	85.0	4	Infertility and volume increase (from 41.5 cm^3^ to 85 in 8 months)	Yes	30.08.2024	43.9/46.6/55.5	59.4	40	2	No
											49.9/40.1/53.5	56.05	96	3	
											41.8/45.4/48.5	48.1	229	2	
4	42	0	0	G3	71.0/60.6/65.8	136.6	3		Yes	07.06.2024	49.9/48.0/49	61.4	48	2	No
											47.2/39.8/43.5	39.1	139	2	
											45.2/36.0/44.7	38.08	299	2	
5	42	0	0	G4	36.6/39.9/38.3	28.0	3		Yes	07.06.2024	29.8/25.5/27.7	10.1	48	2	No
											34.6/33.7/34.1	20.5	139	2	
											30.9/28.2/29.6	13.5	299	2	
6	36	1	1	G4 (isthmus)	36.5/28.0/44.8	23.9	4	Volume increase (from 3.3 cm3 to 23.9 cm3 in 15 months)	Yes	07.10.2024	31.1/33.2/34.2	18.48	31	1	No
											25.0/20.8/27.8	7.56	108	1	
7	35	0	0	G3	43.4/43/40.3	39.37	3	Infertility	Yes	09.11.2024	43.7/38.8/36.1	32.04	40	1	No
											39.8/35.3/34.9	26.67	103	1	
8	35	0	0	G2	54.6/48.6/49.9	68.0	3	Infertility	Yes	09.11.2024	50.3/45.0/47.2	55.9	40	1	No
											36.3/32.4/31.7	19.52	103	1	
9	44	3	2	G0	61.4/35.2/48.3	104.52	4	Heavy bleeding and anemia	Yes	25.11.2024	44.4/33.4/48.6	37.37	24	1	No
											40.0/33.1/32.8	22.73	43	1	
10	52	3	2	G2	21.1/19.6/20.4	8.44	3	Painful intercourse and bleeding	No	21.11.2024	16.8/12.8/15.8	1.77	28	1	No
											12.0/8.2/13.4	0.69	126	1	
11	34	0	0	G2	25.8/23.5/27.5	16.7	4	Volume increase (from 2.02 cm3 la 16.7 cm3 in 12 months)	Yes	24.05.2024	23.4/19.1/21.3	9.51	35	2	No
12	34	0	0	G3	12.9/14.8/13.9	2.6	3	RFA by proximity of another fibroid ablated	Yes	24.05.2024	11.5/7.1/9.3	0.76	35	1	No
13	36	2	0	G3	61.7/40.8/54.2	136.38	4	Infertility	Yes	12.08.2024	52.6/38.0/45.1	47.2	66	1	Transvesical approach (punction of the urinary bladder)
											50.9/38.7/46.2	47.6	85	1	
14	32	2	2	G3	38.5/26.9/38.2	39.5	3	Heavy menstrual bleeding	No	31.05.2024	29.8/26.1/31.0	12.6	38	3	No
											36.2/26.3/34.7	33.03	157	3	
15	35	0	0	G1	66.6/51.7/68.5	123.49	4	Heavy menstrual bleeding and anemia	Yes	07.03.2025	52.4/42.9/52.6	61.91	33	2	No
16	35	0	0	G4	36.6/37.3/40.7	29.09	4	Heavy menstrual bleeding and anemia	Yes	07.03.2025	36.8/27.4/24.6	12.98	33	2	No
17	41	0	0	G3	41.6/31.5/36.5	25.0	3	Heavy menstrual bleeding	No	05.04.2024	27.7/30.0/28.9	12.57	25	1	No
											25.5/22.0/18.5	4.58	440	1	
18	41	0	0	G4	21.3/16.2/19.7	3.56	3	Heavy menstrual bleeding	No	05.04.2024	22.6/15.7/19.1	3.55	25	2	No
											11.0/10.6/10.2	0.59	440	1	
19	38	0	0	G3	30.1/32.7/24.2	12.47	4	Bleeding after intercourse	No	18.10.2024	25.0/24.2/23.0	7.28	37	2	No
											19.8/20.2/20.0	4.14	53	3	
											16.5/11.5/14	1.39	163	1	
20	34	2	0	G3	36.9/38.8/36.6	27.4	4	Infertility	Yes	31.01.2025	37.9/36.0/31.0	22.14	28	1	No
21	43	2	1	G1	52.7/54.6/51.0	76.8	4	Heavy menstrual bleeding	No	28.02.2025	24.8/25.5/23.2	7.68	41	1	Skin burn at the site of the ground pads
											22/21.3/1.8	4.4	89	1	
22	46	3	1	G3	70.9/71.3/70.4	186.34	4	Volume increase and heavy menstrual bleeding	No	07.03.2025	62.6/64.9/61.5	130.82	41	2	No

**Figure 1:**
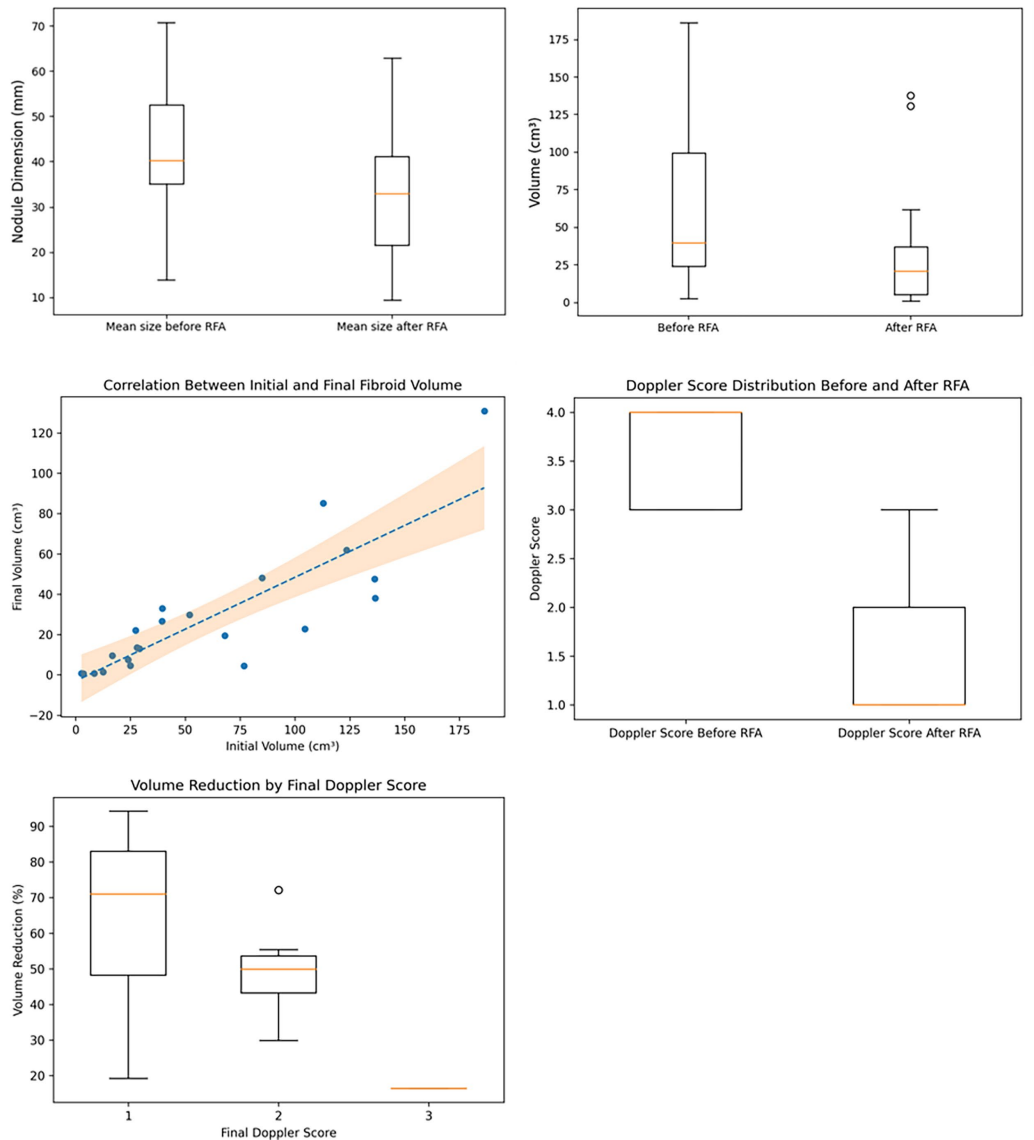
**(A)** Mean size of fibroids before and after RFA; **(B)** Mean fibroid volumes before and after RFA; **(C)** Pearson correlation between baseline and post-treatment fibroid volumes; **(D)** Doppler scores before and after RFA, assessed according to the MUSA criteria; **(E)** Volume reduction by Final Doppler score.

The mean fibroid size before RFA was 42.2 mm (range: 13.8–70.8 mm). After RFA, the mean size decreased to 31.72 mm (range: 9.3–63.0 mm), representing a 24.83% reduction in size (Figure 1A).

The mean fibroid volume before RFA was 60.82 cm^3^ (range: 2.6–186.3 cm^3^). After RFA, the mean volume decreased to 28.3 cm^3^ (range 0.59–130.8 cm^3^), representing a 54.0% decrease (Figure 1B), consistent with observations reported by other authors (6).

A linear regression analysis was performed to evaluate the association between the initial and final fibroid volumes following radiofrequency ablation (RFA), and it showed a Pearson correlation coefficient of *r* = 0.843 (*p*-value < 0.0001). This demonstrates the efficacy of the procedures performed in our unit, aligning us with the current literature (Figure 1C).

The mean Doppler score before RFA was 3.59 (1 fibroid had a score of 2; 9 fibroids had a score of 3, and 12 fibroids had a score of 4). The median Doppler score after ablation was 1.41 (14 fibroids had a Doppler score of 1, 7 fibroids had a score of 2, and 1 fibroid displayed a score of 3 after 5 months). This represents a mean decrease of 2.18 points, or 59.8% reduction from baseline (Figure 1D), which was significantly associated with the percentage of volume reduction after RFA (*r* = −0.476, *p* = 0.025) (Figure 1E).

**Figure 2:**
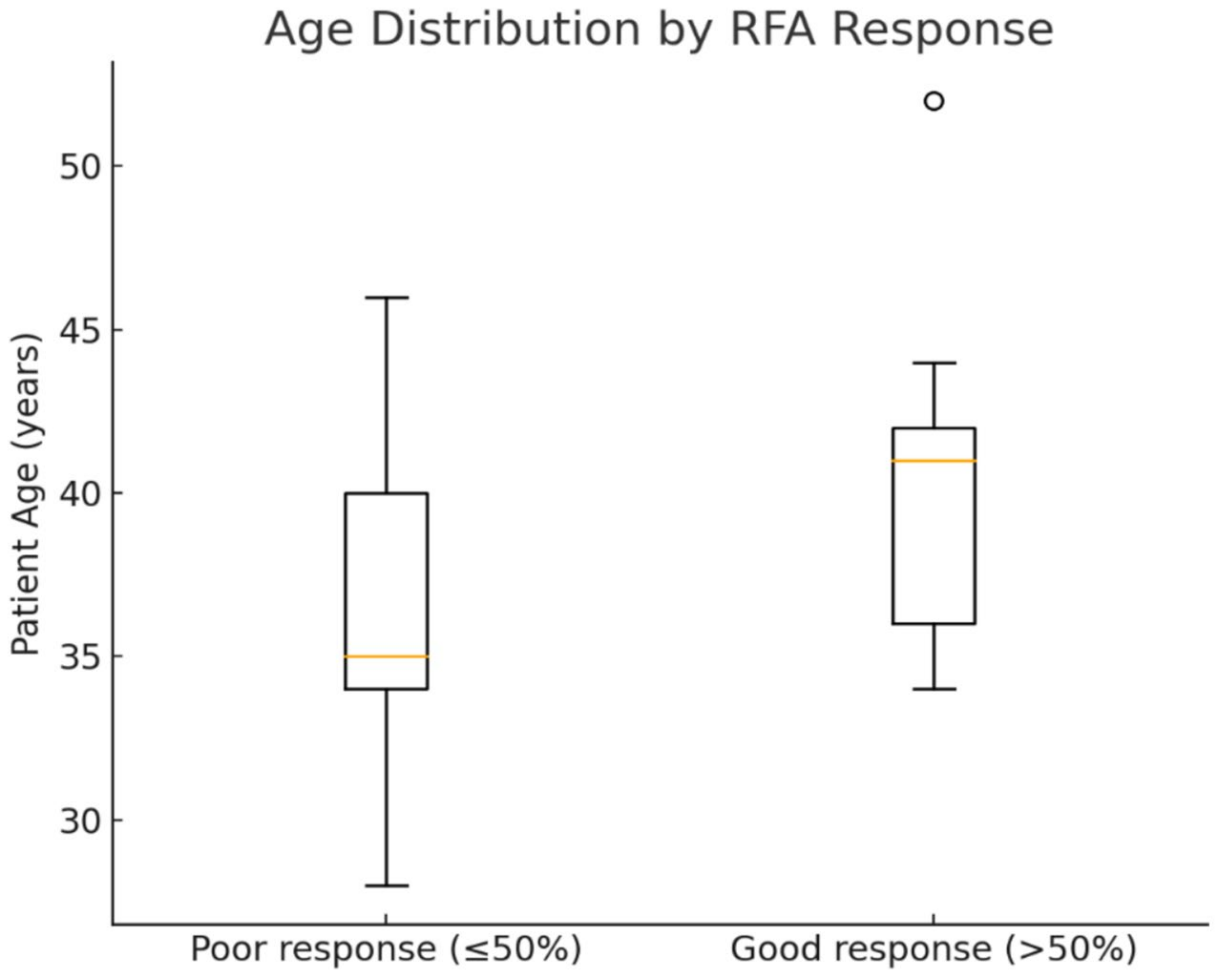
Distribution of patients’ age in good and poor responders to RFA.

There was no statistically significant correlation between age or fibroid location and volume decrease after RFA, although the mean age of good responders was slightly higher (39.9 years) compared to poor responders (36.3 years) (Figure 2).

Patients were followed up for a mean of 141 days after the procedure (range: 28–440 days). Not all patients were able to attend the scheduled visits at 1, 3, 6, and 12 months post-RFA. Nine cases were assessed within the first 3 months, 8 cases within the first 6 months, and only 5 cases were assessed after 6 months. In the subgroup of the nine patients assessed only in the first 3 months after RFA, the mean volume reduction was 56.19%, which may be one of the reasons they did not continue the follow-up.

In terms of complications, two cases (9%) were observed, consistent the data published by other authors (10). In case 13, the procedure was performed via transvesical approach due to the location of the large (136.38 cm^3^) G3 fibroid in the anterior wall. A urinary catheter was placed, and the patient was admitted for observation. Mild hematuria resolved within a few hours, and she was discharged after 24 h with spontaneous urination and clear urine. Case 21 developed second-degree burns on her thighs at the sites where the grounding pads were placed. We believe this event occurred because only two grounding pads were used instead of the four provided in each kit, and the maximum energy applied in her procedure was 140 W. The patient was admitted for observation and wound care for 24 h. The outcome of this case was good, with complete healing of the skin burns within 1 month and a decrease in fibroid volume from 76.8 cm^3^ to 4.4 cm^3^ (96.6%) over 3 months. Case 13 was classified as a Clavien–Dindo type I complication, and case 21 as a type II.

In this series, there were two particular cases that were noteworthy.

**Figure 3:**
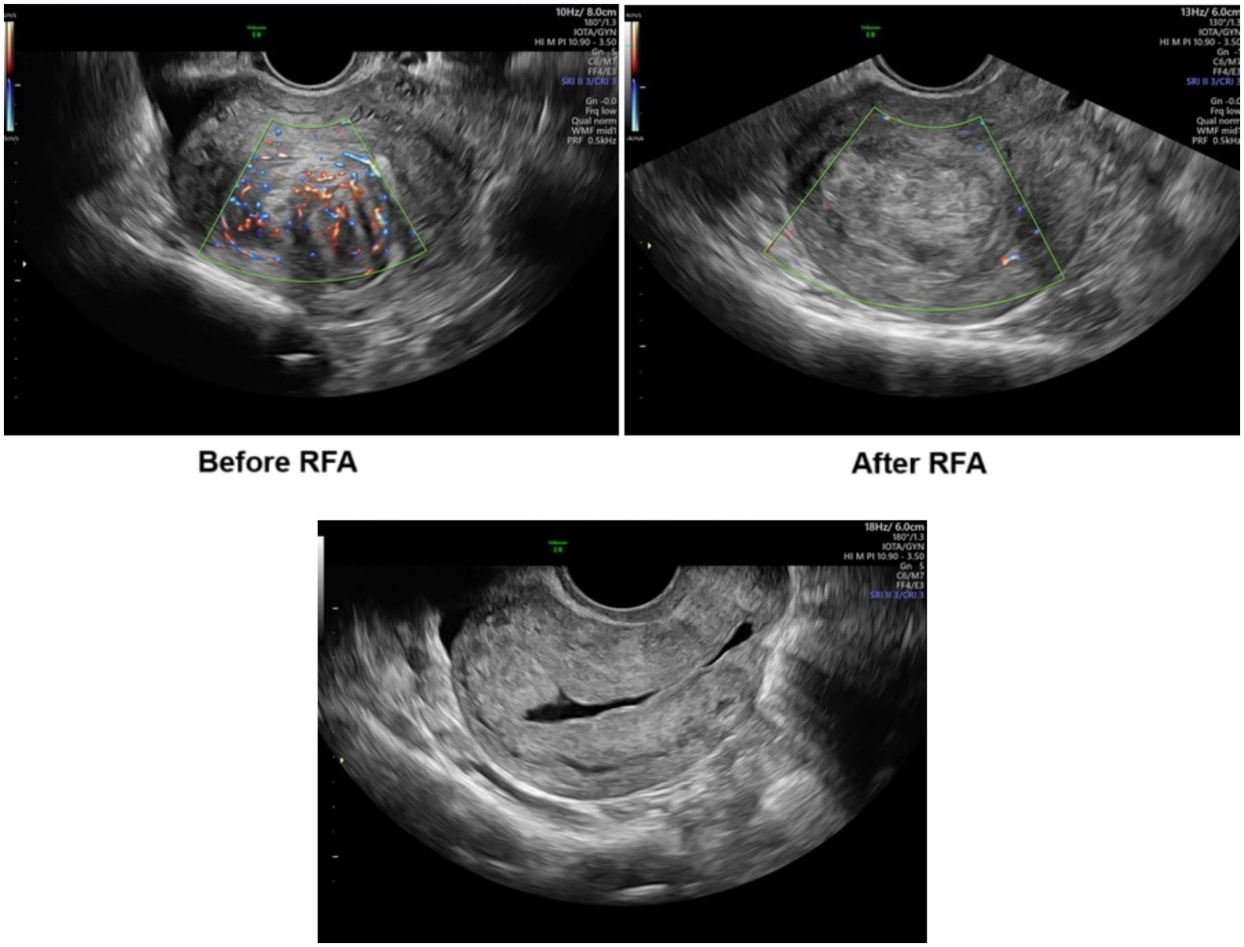
Doppler assessment before RFA (upper-left) and after RFA (upper-right) and ultrasonographic appearance after hysteroscopic myomectomy (bottom).

Case 9 involved a G0 fibroid in a patient presenting with severe anemia that required intravenous hemostatic treatment and blood transfusion. Due to the initial size of the fibroid, its location, and the patient’s hemodynamic status, surgical treatment or embolization of the uterine arteries was initially recommended; however she declined both procedures. As an alternative, she underwent RFA, resulting in cessation of menorrhagia within the first 24 h after the procedure. At the second visit (at 6 weeks), fibroid volume had decreased by 78.2%, and the Doppler score declined from 4 to 1 (Figure 3). No vaginal bleeding was reported during this time, so she was offered a hysteroscopic myomectomy. The intraoperative assessment of the G0 fibroid demonstrated no blood flow, and a complete resection was performed (Figure 3). The patient was discharged the same day.

**Figure 4:**
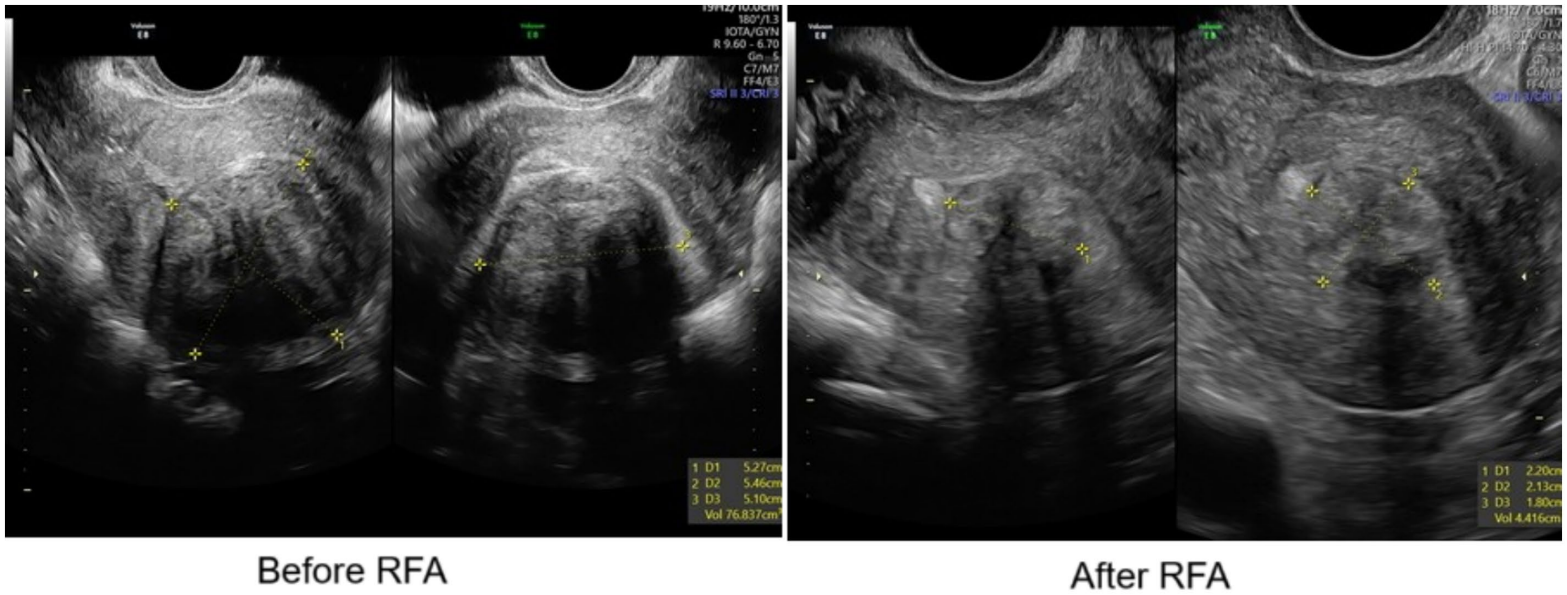
Ultrasound before RFA (left) and after RFA (right).

Case 21 had a G1 fibroid and, despite a Clavien-Dindo type II complication, she experienced the most marked volume reduction: 94% at 3 months after RFA (Figure 4). She was scheduled for a hysteroscopic myomectomy.

**Figure 5:**
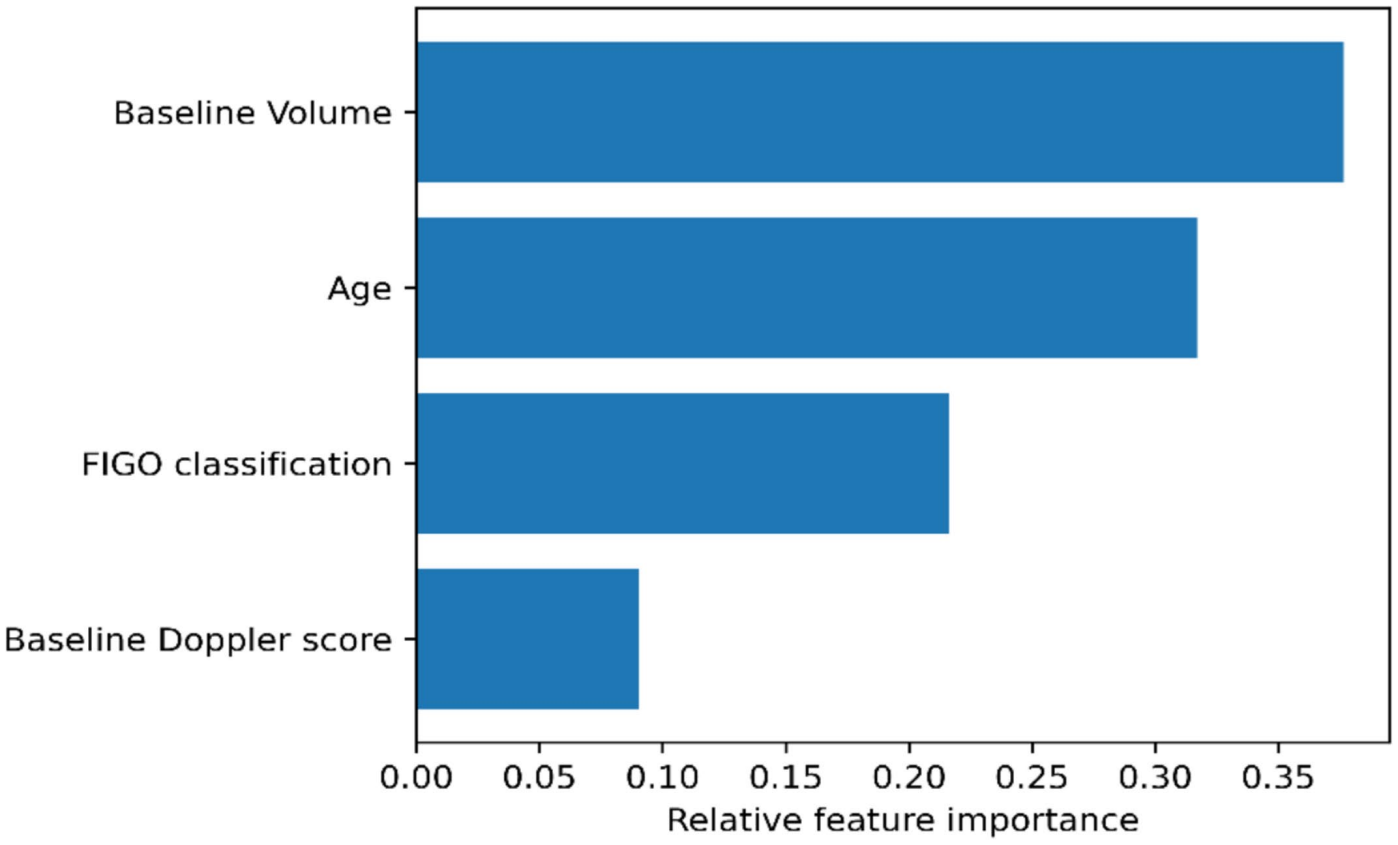
Relative feature importance derived from the Random Forest model for predicting ≥50% fibroid volume reduction.

The predictive analysis utilized a hybrid approach to evaluate factors associated with a volume reduction of ≥50. The Random Forest model, used as an exploratory feature-ranking tool, achieved a mean area under the curve (AUC) of 0.60. Feature importance analysis revealed that baseline volume (0.376) and patient age (0.317) were the most significant contributors to the model’s performance, followed by FIGO stage (0.216) and Doppler score (0.090) (Figure 5).

To provide clinical interpretability, a logistic regression model was constructed to assess the effect of these baseline factors: age, FIGO stage, initial Doppler score, and initial volume, on the treatment outcome. Crude and adjusted odds ratios (OR) were calculated to evaluate both individual and independent effects (Table 3).

**Table 3:** Logistic regression analysis for ≥50% volume reduction after radiofrequency ablation of uterine fibroids.

Predictors	Crude OR [CI 95%]	Adjusted OR [CI 95%]^*^
Age (years)	1.14 [0.94–1.39]	1.16 [0.92–1.46]
FIGO stage (ranging from 0 to 4)	0.80 [0.32–2.00]	0.67 [0.23–1.97]
Doppler score (ranging from 2 to 4)	0.30 [0.05–1.78]	0.37 [0.05–2.79]
Baseline volume of fibroids (mm)	0.99 [0.98–1.01]	0.99 [0.97–1.01]

* ORs were adjusted on all of the predictors presented in this table.

Consistent with the feature importance ranking, the logistic regression analysis showed that none of the four baseline factors reached traditional statistical significance (*p* > 0.05), as indicated by the 95% confidence intervals crossing 1.0.

However, the directional trends complement the machine learning findings:

Age: Identified as a high-importance feature in Random Forest (0.317), age exhibited a slight positive association in the regression model (adjusted OR 1.16), suggesting that older patients might be more likely to achieve the primary outcome.

Doppler Score: Despite its lower relative importance in the Random Forest model (0.090), the logistic regression analysis revealed a strong directional trend (adjusted OR 0.37), indicating that higher vascularity may be associated with a reduced probability of achieving ≥50 volume reduction.

## Discussion

While the majority of studies published focus on volume reduction after RFA and quality of life before and after the procedure, the aim of the study was to prove the concept that data collected systematically and using a standardized protocol could be used to build a prediction model for a good outcome after RFA.

Our hybrid analytical approach demonstrates how machine learning-derived feature importance could be paired with clinical effect estimates. Consistent with previously published data, our series demonstrated a substantial overall mean reduction in fibroid volume (54.0%) and Doppler score (59.8%) following RFA. The strong positive correlation identified between baseline and post-procedural fibroid volume (*r* = 0.843, *p* < 0.0001) confirms the prognostic relevance of initial fibroid size. This was further reinforced by our Random Forest model, which identified baseline volume as the most significant predictor with an importance score of 0.376.

Limited data are available that analyzes the direct association between age and outcome after RFA, but one study published by Santalla-Hernandez et al. (11) reported that increased age was associated with greater fibroid reduction, which is consistent with the trend found in our case series. Our Random Forest analysis ranked age as the second most important feature (0.317), and the logistic regression model showed a corresponding positive trend (adjusted OR = 1.16). Although no statistically significant correlation was found (*p* > 0.05), the high feature ranking in the machine learning model suggests that age may be a critical segmenting factor in larger datasets.

Particular attention should be paid to the Doppler score. While it showed the lowest relative importance in the Random Forest ranking (0.090), the logistic regression analysis provided essential directional insight. The adjusted OR of 0.37 suggests a 63% decrease in the probability of treatment success for each one-unit increase in baseline vascularity. These findings consistently suggest that higher Doppler scores (reflecting increased fibroid vascularization) are associated with a higher risk of treatment failure. The statistically significant reduction in vascularity after RFA correlates well with volume reduction (*r* = −0.476, *p* = 0.025), highlighting Doppler assessment as an independent indicator of efficacy.

The influence of fibroid anatomical location (FIGO classification) remains insufficiently investigated. Our Random Forest model assigned FIGO classification a moderate importance score of 0.216. Despite cautionary guidelines regarding FIGO type G0 or G1 fibroids, we observed excellent outcomes in such cases, with volume reductions exceeding 70%. This allowed for a successful two-step fertility-preserving approach via subsequent hysteroscopic myomectomy. Although no statistically significant correlation was found between FIGO type and response, the feature-importance analysis suggests that it remains a relevant factor for model development. The influence of fibroid anatomical location, as defined by the FIGO classification system, on the efficacy of radiofrequency ablation (RFA) remains insufficiently investigated in the peer-reviewed literature. Landmark trials such as The Fibroid Ablation Study-EU (FAST-EU) (12) and other pivotal prospective studies have demonstrated the overall safety and efficacy of RFA in reducing fibroid volume and improving symptoms; however, none have stratified treatment response based on FIGO classification nor did they report systematic statistical associations between fibroid location and volumetric or clinical outcomes. As such, no current evidence-based guideline defines FIGO type as a predictor of treatment success with RFA. Furthermore, existing device-specific technical recommendations often advise caution when considering treatment of FIGO type G0 or G1 fibroids, citing theoretical concerns related to incomplete coagulation, spontaneous detachment, or expulsion of necrotic tissue. However, these recommendations are largely precautionary and not underpinned by controlled data. We included one FIGO type G0 fibroid and one G1, both of which were fairly large in size, and both exhibited excellent post-procedural outcomes, with volume reductions exceeding 70% and Doppler devascularization. This allowed us to continue the treatment with a uterine-sparing procedure (hysteroscopic myomectomy). In our case series, there was no statistically significant correlation between FIGO type and treatment outcome. This successful RFA followed by hysteroscopic myomectomy highlights the potential for RFA to be offered as a two-step fertility-preserving approach in selected patients with submucosal fibroids, challenging existing cautionary guidelines. The systematic review published by Polin et al. (13) represents the most extensive evaluation of pregnancy outcomes post-RFA (*n* = 923) but does not directly analyze the role of FIGO fibroid classification (G0–G2) in predicting fertility or pregnancy outcomes and most of the nodules were approached laparoscopically, rather than transvaginally (559 vs. 364), suggesting that they were not submucosal. Garza-Leal et al. (14) published a case report of a successful pregnancy with a good outcome after performing RFA for a G1 fibroid measuring 16/16 mm. In this context, the present case series provides original insights suggesting that, under appropriate procedural conditions, submucosal fibroids, even as large as 61.4/35.2/48.3 mm, as in our case 9, may respond favorably, and RFA could be offered as a two-step treatment in infertile patients. In our case, there was no statistically significant correlation between FIGO classification and response to RFA. However, due to the severe class imbalance in our small cohort, this finding cannot be considered definitive and could become an artifact of the limited sample size.

While some studies used the MUSA system to assess the vascularity of the fibroids, they failed to publish the Doppler statistical data regarding the outcome (15). In the vast majority of the studies, Doppler was used during the procedure to ensure a complete ablation of the vessels or as part of the assessment of fibroids at the follow-up visits. Due to this great heterogeneity, systematic reviews or meta-analyses have not been able to draw any conclusion regarding this aspect. Moreover, some studies use atypical or advanced modalities to assess fibroid vascular perfusion that are not easily accessible or reproducible, such as Slow-Flow Doppler provided by Voluson, 3D Power Doppler (16), superb microvascular imaging (17), contrast-enhanced ultrasound (18), and contrast MRI. In our case series, we deployed Doppler techniques available in any ultrasound machine using a standardized score available to any clinician. The statistically significant reduction in vascularity after RFA in our case series correlates well with the volume reduction (*r* = −0.476, *p* = 0.025), in concordance with the data published in the literature, and highlights the utility of Doppler assessment as an independent indicator of treatment efficacy. Its contribution in the prediction model is modest and needs validation in larger datasets.

## Limitations

Sample size and generalizability: The retrospective nature and limited sample size (*N* = 22) indicate that the results must be viewed as preliminary data and proof-of-concept, which restricted the statistical power of both the logistic regression and the Random Forest model.

Imbalance in FIGO Classification: Our cohort exhibited a concentration of cases in specific anatomical locations. This severe class imbalance hinders robust conclusions and likely explains why the FIGO classification did not reach statistical significance in the associative analysis.

Predictive model development: Due to the small sample size, the Random Forest model is highly susceptible to overfitting, as evidenced by the high variance in AUC scores across validation folds—ranging from 0.00 to 1.00 with a mean of 0.60.

Follow-up variability: Follow-up intervals ranged from 28 to 440 days, introducing a methodological challenge in consistently defining the final treatment outcome across the cohort.

Uncontrolled procedural factors: Treatment outcomes are multifactorial, depending on variables not accounted for in our model, such as total energy deployed (60–140 W) and the skill of the physician.

## Conclusion

This study underscores the critical need for structured, uniform, high-quality data acquisition to enable predictive analytics in minimally invasive gynecologic interventions. It also underlines the need for revisiting some of the criteria for RFA, particularly for submucosal fibroids, and this is of particular relevance in scenarios where fertility preservation is of primary concern.

Despite these constraints, this study represents an important proof of concept for the implementation of predictive analytics in minimally invasive fibroid therapy. Future research should be undertaken to validate these findings in larger, multicentric cohorts, in which more advanced analytical methods can be applied. Such developments may ultimately allow the creation of real-time, decision-support guidelines for minimally invasive fibroid therapy in routine clinical practice, potentially enabling direct integration into ultrasound software or patient management systems.

## Data Availability

The original contributions presented in the study are included in the article/supplementary material, further inquiries can be directed to the corresponding author.
